# Efficacy, immunogenicity, and safety of the HPV‐16/18 AS04‐adjuvanted vaccine in Chinese women aged 18–25 years: event‐triggered analysis of a randomized controlled trial

**DOI:** 10.1002/cam4.869

**Published:** 2016-12-20

**Authors:** Feng‐cai Zhu, Shang‐Ying Hu, Ying Hong, Yue‐Mei Hu, Xun Zhang, Yi‐Ju Zhang, Qin‐Jing Pan, Wen‐Hua Zhang, Fang‐Hui Zhao, Cheng‐Fu Zhang, Xiaoping Yang, Jia‐Xi Yu, Jiahong Zhu, Yejiang Zhu, Feng Chen, Qian Zhang, Hong Wang, Changrong Wang, Jun Bi, Shiyin Xue, Lingling Shen, Yan‐Shu Zhang, Yunkun He, Haiwen Tang, Naveen Karkada, Pemmaraju Suryakiran, Dan Bi, Frank Struyf

**Affiliations:** ^1^ Jiangsu Province Center for Disease Prevention and ControlNanjingChina; ^2^ National Cancer Center ‐ Cancer Hospital, Chinese Academy of Medical Sciences (CAMS) & Peking Union Medical College (PUMC)BeijingChina; ^3^ Affiliated Drum Tower Hospital of Nanjing University Medical SchoolNanjingChina; ^4^ Lianshui Center for Disease Prevention and ControlLianshuiChina; ^5^ Jintan Center for Disease Prevention and ControlJintanChina; ^6^ Xuzhou Center for Disease Prevention and ControlXuzhouChina; ^7^ Binhai Center for Disease Prevention and ControlYanchengChina; ^8^ GSK VaccinesShanghaiChina; ^9^ GSK Pharmaceuticals India LtdBangaloreIndia; ^10^ GSK VaccinesWavreBelgium

**Keywords:** Efficacy, HPV‐16/18 AS04‐adjuvanted vaccine, human papillomavirus, immunogenicity, safety

## Abstract

We previously reported the results of a phase II/III, double‐blind, randomized controlled study in Chinese women (NCT00779766) showing a 94.2% (95% confidence interval: 62.7–99.9) HPV‐16/18 AS04‐adjuvanted vaccine efficacy (VE) against cervical intraepithelial neoplasia grade 1 or higher (CIN1+) and/or 6‐month (M) persistent infection (PI) with a mean follow‐up of <2 years, and immunogenicity until 7 months post‐dose 1. Here, we report efficacy and safety results from an event‐triggered analysis with ~3 years longer follow‐up, and immunogenicity until M24. Healthy 18–25‐year‐old women (*N* = 6051) were randomized (1:1) to receive three doses of HPV‐16/18 vaccine or Al(OH)_3_ (control) at M0, 1, 6. VE against HPV‐16/18‐associated CIN2+, and cross‐protective VE against infections with nonvaccine oncogenic HPV types, immunogenicity, and safety were assessed. In the according‐to‐protocol efficacy cohort, in initially seronegative/DNA‐negative women (vaccine group: *N* = 2524; control group: *N* = 2535), VE against HPV‐16/18‐associated CIN2+ was 87.3% (5.3–99.7); VE against incident infection or against 6‐month persistent infection associated with HPV‐31/33/45 was 50.1% (34.3–62.3) or 52.6% (24.5–70.9), respectively. At least, 99.6% of HPV‐16/18‐vaccines remained seropositive for anti‐HPV‐16/18 antibodies; anti‐HPV‐16 and ‐18 geometric mean titers were 1271.1 EU/mL (1135.8–1422.6) and 710.0 EU/ml (628.6–801.9), respectively. Serious adverse events were infrequent (1.7% vaccine group [*N* = 3026]; 2.5% control group [*N* = 3026]). Of the 1595 reported pregnancies, nine had congenital anomalies (five live infants, three elective terminations, one stillbirth) that were unlikely vaccination‐related (blinded data). VE against HPV‐16/18‐associated CIN2+ was demonstrated and evidence of cross‐protective VE against oncogenic HPV types was shown. The vaccine was immunogenic and had an acceptable safety profile.

## Introduction

Cervical cancer is the fourth most common cancer among women worldwide and the second most common female cancer in women aged 15–44 years, with nearly 528,000 women diagnosed with cervical cancer and 266,000 deaths in 2012 1. The burden of cervical cancer is higher in developing countries.

Persistent infection (PI) with high‐risk (HR) human papillomavirus (HPV) types has been established as the major etiological factor for high‐grade cervical intraepithelial neoplasia (CIN) and cervical cancer 2, 3, 4, 5. HPV‐16 and HPV‐18 are the most common HR‐HPV types and are responsible for approximately 70% of cervical cancer cases 2, 6, 7, 8. All sexually active women are at risk of oncogenic HPV infection, with a high prevalence of HPV (12–56%) reported in adolescent and young women, soon after their sexual debut 9, 10.

In China, cervical cancer is a major public health concern, with an estimated 98,900 cases and 30,500 deaths in 2015 11. The reported HPV infection prevalence in China varies from 7% to 32%, depending on the geographical region 12, 13, 14, 15. A recent study of the HPV age‐specific prevalence in Chinese women showed the highest referenced rate of infection with HR‐HPV in the 15–19 years age group, indicating the need for vaccination programs targeting girls before sexual debut and for screening among sexually active women 15. In addition, in a multicenter survey on the age of sexual debut and sexual behavior in Chinese women, a trend toward earlier sexual debut was observed in younger age groups (15–19 and 20–24 years), suggesting that HPV vaccination of girls aged 13–15 years would likely contribute to the prevention of HPV infection and cervical cancer in China 16.

The prophylactic HPV‐16/18 AS04‐adjuvanted vaccine (*Cervarix* ^™^, GSK), is licensed in over 135 countries and was recently approved in China. The vaccine has been shown to be efficacious against high‐grade CIN associated with HPV‐16/18, and irrespective of HPV type in a naïve (i.e., with no evidence of HR‐HPV infection at baseline) population 17, 18; in the total vaccinated cohort of HPV‐naïve women (TVC‐naïve), vaccine efficacy (VE) against CIN1+, CIN2+, and CIN3+ associated with HPV‐16/18 was 96.5% (95% confidence interval [CI] 91.6–98.9), 99.0% (94.2–100), and 100% (85.5–100), and VE against these endpoints irrespective of HPV DNA was 50.3% (40.2–58.8), 64.9% (52.7–74.2), and 93.2% (78.9–98.7), respectively 17. The safety, immunogenicity, and efficacy of the vaccine have been studied in several large trials in women aged 15–25 years in various countries outside China 17, 18, 19, 20, 21, 22, 23, 24, 25, 26, 27, 28, 29. In a long‐term efficacy follow‐up study up to 6.4 years in women aged 15–25 years at the time of first vaccination, VE against incident infection, or 6‐ or 12‐month (M) PI with HPV‐16/18 in the according‐to‐protocol (ATP) cohort for efficacy (ATP‐E) was 95.3% (87.4–98.7), 100% (90.0–100), and 100% (81.8–100), respectively, and VE against HPV‐16/18‐associated CIN1+ and CIN2+ in the TVC for efficacy (TVC‐E) was 100% (73.4–100) and 100% (51.3–100), respectively 19; sustained anti‐HPV‐16/18 antibody levels remaining several folds above natural infection levels were also observed 27. However, to date, the efficacy of HPV vaccination against CIN2+ has not been reported among the Chinese population.

In this manuscript, we report the results from an extended follow‐up of a primary, phase II/III study, evaluating the efficacy of the HPV‐16/18 AS04‐adjuvanted vaccine against CIN1+ and/or 6M PI in Chinese women aged 18–25 years; results up to approximately 24 months post‐dose 1 have been previously reported 30. The primary study objective was to demonstrate the efficacy of the HPV‐16/18 AS04‐adjuvanted vaccine in the prevention of histopathologically confirmed CIN1+ and/or 6M PI associated with HPV‐16/18, overall and according to initial (prevaccination) HPV‐16/18 serostatus. The secondary objectives included the evaluation of VE in the prevention of histopathologically confirmed CIN2+ associated with HPV‐16/18, cross‐protective VE against infections associated with nonvaccine oncogenic HPV types, vaccine immunogenicity, and safety.

As the primary study endpoint of VE against CIN1+ and/or 6M PI was met within 24 months of the follow‐up time post‐dose 1, the current extended follow‐up focuses on the evaluation of VE against CIN2+ associated with HPV‐16/18 up to 72 months post‐dose 1. Here, we report the efficacy and safety results from an event‐triggered analysis with a mean follow‐up time of 57 months post‐dose 1. Immunogenicity results obtained in a subset of participants up to 24 months post‐dose 1 are also presented.

## Materials and Methods

### Study design and participants

**Figure 1:**
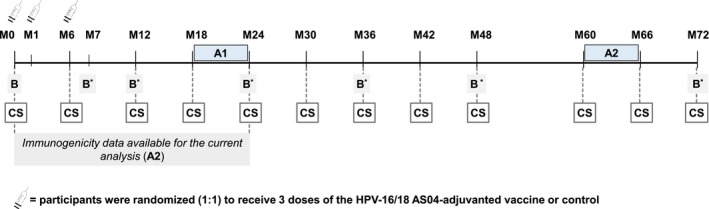
Study design. M, month; B, blood sampling; CS, cervical sample. A1:event‐triggered analysis with a mean follow‐up of ~21 months post‐dose 1 30. A2:current analysis: event‐triggered analysis with a mean follow‐up time of ~57 months post‐dose 1 (total vaccinated cohort for efficacy). *only for immunogenicity subset.

This study is a multicenter, double‐blind, randomized, controlled extended follow‐up of a primary study (NCT00779766) conducted in four centers in China. Participants were randomized (1:1) to receive three doses of the HPV‐16/18 AS04‐adjuvanted vaccine or aluminium hydroxide [Al(OH)_3_] at M0, 1, 6, as previously described 30. The enrolment started in October 2008 and study follow‐up was extended from 24 to 72 months. The last visits included in this event‐triggered analysis, M60 or M66, occurred in 2014 for majority of women. The overall study design is summarized in Figure 1.

Study participants were healthy Chinese women aged 18–25 years at the time of first vaccination who provided written informed consent prior to enrolment, and who were of nonchildbearing potential or who practiced adequate contraception for 30 days prior to vaccination and agreed to continue such precautions up to 2 months after completion of the vaccination. The main exclusion criteria included pregnancy, immunosuppressive/immunodeficient condition, and previous HPV vaccination. Details of inclusion/exclusion study criteria, the ethical statement, and vaccine composition have been described in the primary publication 30. This study has been registered at www.clinicaltrials.gov (NCT00779766).

### Randomization and blinding

The treatment allocation at the investigator site was performed using a central randomization system on Internet (SBIR). This study is performed in a double‐blinded manner and will be maintained blinded until the study end. To reduce the risk of unblinding, all analyses were conducted by an external statistician and neither the investigators nor the study team had access to data that could potentially lead to unblinding of individual subjects’ treatment allocation.

### Efficacy and immunogenicity assessments

The timepoints of blood and cervical sampling are shown in Figure 1. Of note, at the time of the current event‐triggered analysis, immunogenicity data were available only up to M24. Cytology, histopathology, HPV DNA testing, and serological assays were performed in local laboratories as previously described 30.

Cervical samples for HPV DNA testing and cervical cytology samples were collected at each study visit (Fig. 1). Cervical cytology was performed using the ThinPrep PapTest (Cytyc Corporation, Boxborough, MA); cytology specimens were evaluated according to the Bethesda 2001 classification system for reporting cervical cytology diagnoses.

Biopsy and excisional treatment specimens were fixed in buffered formalin and analyzed by a panel of three expert gynecological pathologists. A fourth pathologist coordinated the independent and blind review process, and ensured that agreement on the location and grade of the lesion in the tissue was obtained between at least two members of the panel. An Endpoint Committee composed of two independent external experts was responsible for reviewing blinded relevant clinical and laboratory data to make final CIN case assignments.

HPV DNA testing was performed by polymerase chain reaction (PCR), using specific SPF10 primers amplifying a 65‐nucleotide region of the *HPV L1* gene for most of the known HPV isolates. HPV‐positive specimens were typed by reverse hybridization line probe assay, using 28 HPV‐specific hybridization probes enabling detection of 14 oncogenic and 11 nononcogenic HPV types. All HPV‐positive samples were also tested by HPV‐16‐ and HPV‐18‐specific PCRs 31.

Antibody responses against HPV‐16 and HPV‐18 were quantified by an enzyme linked‐immunosorbent assay (ELISA) using either HPV‐16 or HPV‐18 virus‐like particles as coating antigens. The cut‐off values were 8 ELISA units (EU)/mL for anti‐HPV‐16 and 7 EU/mL for anti‐HPV‐18.

### Safety assessment

Serious adverse events (SAEs), medically significant conditions (MSCs), new‐onset of chronic diseases (NOCDs), new‐onset of autoimmune diseases (NOADs), and pregnancy outcomes were assessed throughout the study.

MSCs were defined as: adverse events (AEs) prompting emergency room or physician visits that were not related to common diseases or not routine visits for physical examination or vaccination, or SAEs that were not related to common diseases. Common diseases included: upper respiratory infections, sinusitis, pharyngitis, gastroenteritis, urinary tract infections, cervicovaginal yeast infections, menstrual cycle abnormalities, and injury.

Pregnancies around vaccinations were defined as pregnancies in women for whom the last menstrual period occurred between 30 days before and 45 days after vaccination.

### Statistical methods

#### Efficacy analyses

The primary objective of the study was previously assessed in an event‐triggered analysis when 17 cases of CIN1+ and/or 6M PI associated with HPV‐16/18 were observed (up to M24 with a mean follow‐up time of approximately 21 months post‐dose 1) 30.

The study was extended up to M72 to allow for the evaluation of VE against CIN2+ lesions associated with HPV‐16/18. This event‐triggered analysis of efficacy was performed when at least nine cases of CIN2+ associated with HPV‐16/18 infection were observed in the ATP cohort for efficacy (ATP‐E) in DNA‐negative and seronegative participants for the corresponding HPV type at baseline. If efficacy against this endpoint was demonstrated before M72, that is, the lower limit (LL) of 95% CI around the VE of CIN2+ associated with HPV‐16/18 was above 0, the “end of study rule” applied, and participants were to end their study participation after a last study visit to complete all study conclusion procedures.

The sample size, study power for the primary combined endpoint (histopathologically confirmed CIN1+ and/or 6M PI associated with HPV‐16/18), secondary endpoint (histopathologically confirmed CIN2+ associated with HPV‐16/18), as well as the study cohorts were previously described 30.

Assuming VE of 90% for the secondary endpoint of CIN2+ associated with HPV‐16/18 20, 21, it was calculated that nine cases (one in the vaccine group and eight in the control group) of CIN2+ were required to have at least 81% power to obtain a significant result (defined as LL of the 95% CI for VE above 0). Based on an estimated yearly rate of 0.08% for CIN2+ associated with HPV‐16/18 in the control group, it was expected that nine cases of CIN2+ will have accrued by the time of M72 analysis, assuming that a total of 2100 subjects would be evaluable in ATP‐E at M72.

The primary efficacy analyses were performed on the ATP‐E in participants who were seronegative (by ELISA) at M0 and DNA negative (by PCR) at M0 and M6 for the HPV type considered in the analysis. Additional analyses based on the TVC‐E were performed to complement the ATP‐E analysis. Analyses of the primary endpoint, and cytological and histopathological endpoints associated with HPV‐16 or HPV‐18 in the ATP‐E were stratified according to initial (M0) HPV‐16 or 18 serostatus (as determined by ELISA). For all serostratified secondary and exploratory efficacy analyses, the principal analysis was performed on women who were seronegative (by ELISA) prior to vaccination for the HPV type considered in the analysis.

The ATP‐E cohort included all evaluable participants (i.e., those who met all eligibility criteria, received three doses of the study vaccine or control, complied with the procedures defined in the protocol, with no elimination criteria) for whom efficacy data were available, and who had a normal or low‐grade cytology (negative or atypical squamous cells of undetermined significance [ASC‐US] or low‐grade squamous intraepithelial lesions [LSIL]) at M0. The TVC‐E included all vaccinated participants, who received at least one dose of the study vaccine or control, for whom efficacy data were available, and who had a normal or low‐grade cytology (negative or ASC‐US or LSIL) at M0.

VE was calculated using a conditional exact method 32, as previously described 30. Results were considered statistically significant if the LL of the 95% CI for VE was above 0.

Case counting started on the day after the third dose (ATP‐E cohort) or on the day after the first dose (for the TVC‐E cohort) and ended at the time of an endpoint event.

#### Immunogenicity and safety analyses

Immunogenicity analyses were performed in a subset of participants recruited exclusively from one site. Primary analyses of immunogenicity were performed on the ATP cohort for immunogenicity (ATP‐I) which included all evaluable participants in the immunogenicity subset (i.e., those meeting all eligibility criteria, complying with the procedures defined in the protocol, with no elimination criteria during the study) for whom immunogenicity data were available. Participants who acquired either HPV‐16 or HPV‐18 infection during the study were excluded from the ATP‐I.

Seropositivity was defined as a titer ≥ the cut‐off value. Seroconversion was defined as the appearance of antibodies in the serum of participants seronegative before vaccination.

Geometric mean titer (GMT) calculations were performed by taking the antilog of the mean of the log titer transformations. Antibody titers below the cut‐off of the assay were given an arbitrary value of half the cut‐off for GMT calculation.

Safety analyses were performed on the TVC, which included all participants who received at least one vaccine dose.

## Results

### Study participants

**Figure 2:**
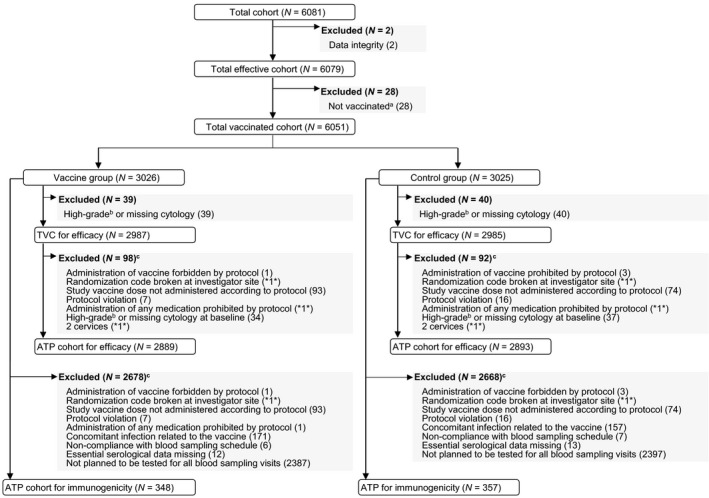
Participant flow. N, number of subjects included in each group; *n*, number present in one group only and duplicated to avoid unblinding of ongoing study; ATP, according‐to‐protocol; TVC, total vaccinated cohort. ^a^Study vaccine dose not administered but subject number allocated. ^b^Atypical squamous cells cannot exclude high‐grade squamous intraepithelial lesions, high‐grade squamous intraepithelial lesions, atypical glandular cells, or malignancy. ^c^Subjects may have more than one elimination code assigned ATP cohort for efficacy included participants who were seronegative (by ELISA) at Month 0 and DNA negative (by polymerase chain reaction [PCR]) at Month 0 and 6 for the HPV type considered in the analysis.

A total of 6081 women were enrolled in the study; 6051 were included in the TVC (3026 in the vaccine group and 3025 in the control group), and 5972 were included in the TVC‐E (2987 in the vaccine group and 2985 in the control group). Of the 5972 women included in TVC‐E, 5782 (2889 in the vaccine group and 2893 in the control group) were included in the ATP‐E; a subset of 705 women (348 in the vaccine group and 357 in the control group) were included in the ATP‐I at M24 (Fig. 2). Demographic characteristics were previously presented 30. Baseline HPV DNA prevalence and seroprevalence were reported in a separate manuscript 33.

The mean follow‐up time was approximately 57 months for the TVC‐E (counting from M0) and approximately 52 months for the ATP‐E (counting from M6).

### Efficacy

The main objective of the current follow‐up of VE against histopathologically confirmed CIN2+ associated with HPV‐16/18 was met, as the LL of the 95% CI was above 0. In the ATP‐E, for initially HPV DNA‐negative and seronegative participants, VE against histopathologically confirmed CIN2+ associated with HPV‐16/18 was 87.3% (95% CI: 5.3–99.7), with one case in the vaccine group and eight cases in the control group; a high VE (88.7%; 18.4–99.7) against CIN2+ was also shown in the TVC‐E (Table 1). Of note, the CIN2+ case observed in the vaccine group in the ATP‐E was diagnosed as CIN3+ and multiple oncogenic HPV infections were detected in the cervical specimen (Table 1).

**Table 1:** Vaccine efficacy against cervical intraepithelial neoplasia and persistent infection associated with HPV‐16 and/or HPV‐18 in women who were HPV DNA‐negative and seronegative at baseline for the corresponding HPV type (ATP‐E and TVC‐E)

HPV‐16/18 Endpoint	ATP‐E			TVC‐E		
	Vaccine	Control	% VE (95% CI)	Vaccine	Control	% VE (95% CI)
	*N*/*n*			*N*/*n*		
CIN2+	2524/1	2535/8	87.3 (5.3–99.7)	2567/1	2587/9	88.7 (18.4–99.7)
CIN1+	2524/1	2535/15	93.2 (56.1–99.8)	2567/2	2587/17	88.0 (49.6–98.7)
CIN1+/6M PI	2524/2	2535/60	96.7 (87.4–99.6)	2567/5	2587/78	93.6 (84.4–98.0)
6M PI	2480/2	2488/54	96.3 (85.9–99.6)	2551/4	2571/71	94.4 (84.9–98.5)
12M PI	2425/1	2455/32	96.9 (81.1–99.9)	2516/3	2536/41	92.6 (76.9–98.5)

ATP‐E, according‐to‐protocol cohort for efficacy; TVC‐E, total vaccinated cohort for efficacy; Vaccine, women who received up to three doses of the HPV‐16/18 AS04‐adjuvanted vaccine; Control, women who received up to three doses of aluminium hydroxide; *N,* number of participants included in each group; *n*, number of cases; VE, vaccine efficacy; 95% CI, 95% confidence interval; CIN2+, cervical intraepithelial neoplasia grade 2 and above; CIN1+/6M PI, cervical intraepithelial neoplasia grade 1 and above and/or 6‐month persistent infection.

CIN2+ was defined as CIN2, CIN3, low‐grade cervical glandular intraepithelial neoplasia (LCGIN), high‐grade cervical glandular intraepithelial neoplasia (HCGIN), adenocarcinoma in situ (AIS), or invasive cervical cancer.

CIN1+ was defined as CIN1, CIN2, CIN3, LCGIN, HCGIN, AIS, or invasive cervical cancer.

6M PI with HPV‐16/18 was defined as at least two positive HPV DNA PCR assays for the same viral genotype with no negative DNA sample between the two positive DNA samples, over at least 150 days.

For participants who were HPV DNA‐negative for the corresponding HPV type at M0 and M6, regardless of their initial HPV serostatus, VE against histopathologically confirmed CIN2+ associated with HPV‐16/18 was 90.0% (29.4–99.8) in the ATP‐E, with one case in the vaccine group and 10 cases in the control group. In the TVC‐E, VE was 83.2% (24.7–98.2) (Table S1).

In the ATP‐E, for initially HPV DNA‐negative and seronegative participants, VE for the primary combined endpoint (CIN1+ and/or 6M PI associated with HPV‐16/18) was 96.7% (87.4–99.6). Two cases were associated with HPV‐16/18 in the vaccine group and 60 cases were associated with HPV‐16/18 in the control group. High VE for the primary endpoint was also shown in the TVC‐E (93.6%; 84.4–98.0) (Table 1). In the ATP‐E, for participants who were HPV DNA‐negative for the corresponding HPV type at baseline (i.e., M0) and M6, regardless of their initial HPV serostatus, VE for the primary combined endpoint was 97.5% (90.5–99.7), with two cases reported in the vaccine group and 78 cases in the control group. VE was 93.0% (85.1–97.3) in the TVC‐E (Table S1).

In the ATP‐E, for participants initially HPV DNA‐negative at M0 and 6, regardless of their initial HPV serostatus, a significant VE against incident infection was shown for HPV‐31 (55.6% [32.2–71.5]), HPV‐33 (42.0% [13.1–61.7]), and HPV‐45 (52.3% [14.4–74.4]), while VE against 6M PI or 12M PI was significant for HPV‐31 (65.6% [27.3–85.0] and 81.2% [34.4–96.5]), but not for HPV‐33 (47.9% [−5.9–75.5] and 10.6% [−161.2–70.0]) or HPV‐45 (11.9% [−178.0–72.8] and −152.3% [−2549.9–58.7]) (Table 2). VE against incident infection with combined HPV‐31/33/45 was 50.1% (34.3–62.3), with 80 cases in the vaccine group and 159 cases in the control group; VE in the TVC‐E was 46.8% (32.4–58.3). In the ATP‐E, VE against 6M PI and 12M PI associated with HPV‐31/33/45 was 52.6% (24.5–70.9) and 44.2% (−8.6–72.4), respectively, and in the TVC‐E, 41.9% (15.8–60.4) and 43.1% (0.2–68.3), respectively (Table 2).

**Table 2:** Vaccine efficacy against incident or persistent infection with oncogenic HPV types, individually or in combination, in women HPV DNA‐negative at baseline, regardless of initial serostatus (ATP‐E and TVC‐E)

Endpoint	HPV type	ATP‐E			TVC‐E		
		Vaccine	Control	% VE (95% CI)	Vaccine	Control	% VE (95% CI)
		*N*/*n*			*N*/*n*		
Incidentinfection	HPV‐16	2708/26	2710/127	79.6 (68.7–87.2)	2807/39	2835/150	74.0 (62.9–82.2)
	HPV‐18	2770/18	2768/74	75.8 (59.0–86.4)	2872/28	2891/92	69.6 (53.2–80.8)
	HPV‐31	2776/33	2770/74	55.6 (32.2–71.5)	2875/42	2897/97	56.8 (37.3–70.6)
	HPV‐33	2769/40	2771/69	42.0 (13.1–61.7)	2872/55	2897/88	37.3 (11.2–56.1)
	HPV‐35	2795/49	2791/55	10.5 (−34.1–40.3)	2890/53	2908/67	20.4 (−15.9–45.6)
	HPV‐39	2748/136	2760/121	−13.7 (−46.5–11.7)	2858/157	2888/151	−5.1 (−32.3–16.5)
	HPV‐45	2783/18	2789/38	52.3 (14.4–74.4)	2885/28	2908/50	43.6 (8.6–65.8)
	HPV‐51	2742/152	2743/187	18.4 (−1.6–34.6)	2862/187	2867/209	10.1 (−10.0–26.6)
	HPV‐52	2656/227	2652/264	13.4 (−3.8–27.8)	2798/291	2806/321	8.6 (−7.4–22.3)
	HPV‐56	2772/93	2767/106	12.2 (−17.2–34.2)	2874/107	2887/122	12.0 (−15.0–32.8)
	HPV‐58	2761/77	2756/99	22.1 (−6.1–42.9)	2865/90	2879/121	25.4 (1.1–43.8)
	HPV‐59	2803/47	2784/62	24.5 (−12.1–49.4)	2898/52	2905/74	29.8 (−1.4–51.7)
	HPV‐66	2769/96	2752/111	13.5 (−14.8–34.8)	2877/116	2877/131	11.3 (−14.8–31.5)
	HPV‐68	2764/91	2772/95	3.6 (−29.9–28.5)	2875/114	2896/113	−1.7 (−33.1–22.3)
	HPV‐31/33/45	2812/80	2811/159	50.1 (34.3–62.3)	2904/108	2921/201	46.8 (32.4–58.3)
	HPV‐16/18/31/33/45	2812/120	2812/329	64.6 (56.2–71.5)	2904/168	2921/390	58.2 (49.8–65.3)
	HRW‐HPV	2812/692	2812/800	14.4 (5.1–22.8)	2904/811	2921/914	11.7 (2.9–19.8)
	HR‐HPV	2812/710	2812/862	19.3 (10.8–27.1)	2904/835	2921/976	15.5 (7.2–23.0)
6M PI	HPV‐16	2663/2	2655/51	96.1 (85.1–99.5)	2746/3	2761/61	95.1 (84.9–99.0)
	HPV‐18	2723/0	2710/21	100.0 (80.8–100.0)	2807/2	2817/32	93.7 (75.5–99.3)
	HPV‐31	2731/10	2714/29	65.6 (27.3–85.0)	2812/15	2824/39	61.4 (28.4–80.2)
	HPV‐33	2722/13	2712/25	47.9 (−5.9–75.5)	2808/22	2823/32	30.7 (−23.1–61.6)
	HPV‐35	2747/17	2734/18	5.3 (−94.8–54.1)	2826/17	2834/24	28.7 (−38.3–64.1)
	HPV‐39	2701/47	2702/41	−15.7 (−80.4–25.5)	2794/56	2816/54	−5.0 (−55.6–29.0)
	HPV‐45	2735/7	2733/8	11.9 (−178.0–72.8)	2820/12	2834/15	19.3 (−84.6–65.5)
	HPV‐51	2699/59	2685/58	−2.5 (−49.8–29.9)	2799/74	2795/71	−4.9 (−47.4–25.3)
	HPV‐52	2612/109	2602/97	−13.8 (−51.1–14.3)	2737/144	2734/120	−21.8 (−56.4–5.1)
	HPV‐56	2726/32	2709/36	11.0 (−47.4–46.5)	2810/42	2815/41	−3.2 (−62.7–34.5)
	HPV‐58	2715/32	2699/40	19.9 (−30.7–51.3)	2800/39	2807/51	23.2 (−18.9–50.7)
	HPV‐59	2755/15	2726/15	0.2 (−119.1–54.5)	2833/18	2830/21	14.0 (−69.4–56.8)
	HPV‐66	2723/31	2694/29	−6.7 (−83.5–37.8)	2812/38	2804/33	−15.5 (−90.0–29.5)
	HPV‐68	2720/28	2714/30	6.2 (−62.6–46.0)	2810/41	2823/39	−6.1 (−68.8–33.3)
	HPV‐31/33/45	2764/28	2752/59	52.6 (24.5–70.9)	2839/47	2846/81	41.9 (15.8–60.4)
	HPV‐16/18/31/33/45	2764/30	2753/126	76.5 (64.8–84.8)	2839/52	2846/164	68.8 (57.1–77.6)
	HRW‐HPV	2764/319	2753/357	10.4 (−4.5–23.2)	2839/406	2846/432	5.1 (−8.9–17.3)
	HR‐HPV	2764/320	2753/400	20.6 (7.8–31.6)	2839/408	2846/482	15.5 (3.4–26.2)
12M PI	HPV‐16	2604/1	2620/29	96.5 (79.1–99.9)	2712/2	2720/36	94.4 (78.3–99.4)
	HPV‐18	2664/0	2672/8	100.0 (41.2–100.0)	2772/2	2773/14	85.7 (37.5–98.4)
	HPV‐31	2671/3	2676/16	81.2 (34.4–96.5)	2777/3	2779/20	84.9 (49.2–97.1)
	HPV‐33	2663/8	2675/9	10.6 (−161.2–70.0)	2773/12	2778/11	−10.1 (−175.4–55.5)
	HPV‐35	2686/11	2695/9	−22.9 (−235.5–53.7)	2790/11	2790/14	20.9 (−87.5–67.5)
	HPV‐39	2641/25	2664/22	−14.8 (−113.6–37.9)	2758/32	2772/28	−15.8 (−99.6–32.5)
	HPV‐45	2674/5	2694/2	−152.3 (−2549.9–58.7)	2784/7	2791/6	−17.7 (−323.9–66.1)
	HPV‐51	2639/28	2647/24	−17.6 (−112.0–34.3)	2765/38	2750/31	−23.3 (−104.9–25.3)
	HPV‐52	2553/70	2569/63	−12.4 (−60.5–21.2)	2703/96	2694/74	−31.3 (−80.2–4.1)
	HPV‐56	2665/14	2672/11	−27.9 (−211.3–46.1)	2775/16	2770/13	−23.9 (−179.9–44.1)
	HPV‐58	2656/15	2661/18	16.4 (−75.8–60.8)	2766/19	2764/22	13.1 (−68.2–55.5)
	HPV‐59	2694/7	2687/2	−250.2 (−3355.0–33.3)	2797/9	2785/4	−125.9 (−904.0–36.9)
	HPV‐66	2662/17	2657/10	−70.3 (−316.2–26.4)	2777/22	2759/13	−69.8 (−267.0–18.2)
	HPV‐68	2659/12	2675/12	−0.8 (−145.3–58.6)	2776/18	2779/19	4.6 (−92.1–52.8)
	HPV‐31/33/45	2703/15	2713/27	44.2 (−8.6–72.4)	2803/21	2801/37	43.1 (0.2–68.3)
	HPV‐16/18/31/33/45	2703/16	2714/63	74.7 (55.7–86.4)	2803/25	2801/84	70.4 (53.3–81.9)
	HRW‐HPV	2703/192	2714/188	−3.1 (−26.8–16.1)	2803/249	2801/235	−7.5 (−29.0–10.4)
	HR‐HPV	2703/192	2714/215	10.4 (−9.3–26.7)	2803/250	2801/269	6.5 (−11.5–21.6)

ATP‐E, according‐to‐protocol cohort for efficacy; TVC‐E, total vaccinated cohort for efficacy; Vaccine, women who received up to three doses of the HPV‐16/18 AS04‐adjuvanted vaccine; Control, women who received up to three doses of aluminium hydroxide; M, month; PI, persistent infection; *N*, number of participants included in each group; *n*, number of cases; VE, vaccine efficacy; 95% CI, 95% confidence interval; HRW‐HPV, high‐risk (oncogenic) HPV types without HPV‐16 or HPV‐18: HPV‐31, 33, 35, 39, 45, 51, 52, 56, 58, 59, 66, and 68; HR‐HPV, high‐risk (oncogenic) HPV types: HPV‐16, 18, 31, 33, 35, 39, 45, 51, 52, 56, 58, 59, 66, and 68.

### Immunogenicity

The HPV‐16/18 baseline serostatus was similar in both groups. The majority (63.3%) of women were seronegative for both HPV‐16 and HPV‐18 at study start (63.5% in the vaccine group and 63.0% in the control group). In the vaccine group, 11.8% of women were seropositive for both HPV‐16 and HPV‐18 antibodies, 18.7% were seropositive for HPV‐16 alone, and 6.0% were seropositive for HPV‐18 alone. In the control group, 9.2% of women were seropositive for both HPV‐16 and HPV‐18 antibodies, 22.4% were seropositive for HPV‐16 alone, and 5.3% were seropositive for HPV‐18 alone.

**Figure 3:**
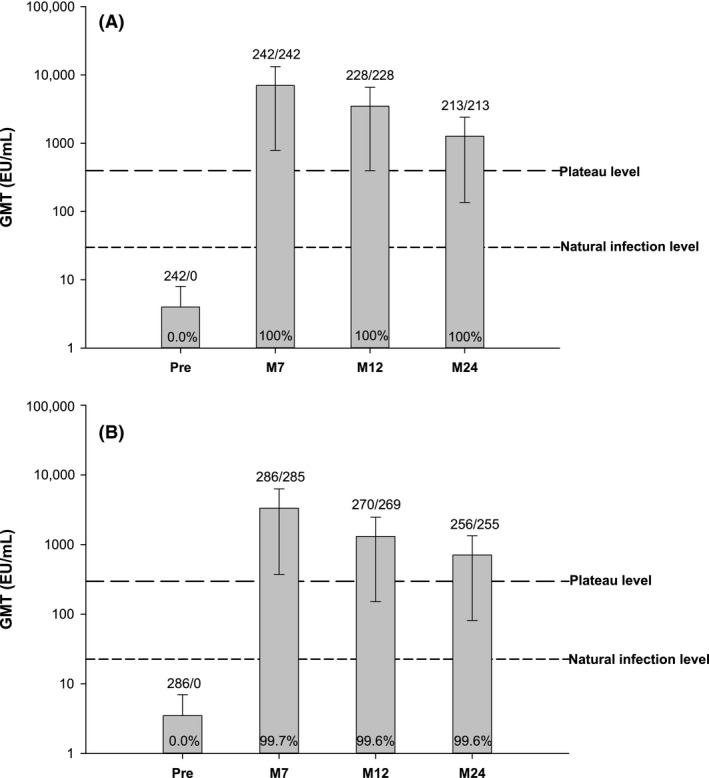
Pre‐ and postvaccination (**A**) anti‐HPV‐16 and (**B**) anti‐HPV‐18 geometric mean titers in initially seronegative women from the vaccine group (ATP cohort for immunogenicity). GMT, geometric mean titer; EU, ELISA units; Pre, prevaccination; M, month; ATP, according‐to‐protocol. Plateau level: GMTs of women aged 15–25 years at months 45–50 after the first vaccine dose (total vaccinated cohort) from a previous long‐term efficacy study (HPV‐007); GMTs were (A) 397.8 EU/mL and (B) 297.3 EU/mL 24. Natural infection level: GMTs of women who were (A) HPV‐16 or (B) HPV‐18 DNA‐negative and seropositive at baseline (i.e., who had cleared a natural infection); GMTs were (A) 29.8 EU/mL and (B) 22.6 EU/mL 15, 16. At the time of this event‐triggered analysis, immunogenicity results were only available up to the M24 timepoint. The percentages of seropositive women for the respective HPV type at each timepoint of assessment are indicated within each graph bar. The numbers indicated above each graph bar represent the number of women with prevaccination results available/number of women with titer ≥ the ELISA cut‐off (8 EU/mL for anti‐HPV‐16 or 7 EU/mL for anti‐HPV‐18). The error bars represent 95% confidence intervals.

At M24, all women from the vaccine group remained seropositive for anti‐HPV‐16 antibodies, and 99.6% of women were seropositive for anti‐HPV‐18 antibodies (Fig. 3). In the control group, 17.3% and 26.0% of women were seropositive for anti‐HPV‐16 and anti‐HPV‐18 antibodies, respectively.

At M24, in initially seronegative women from the vaccine group, anti‐HPV‐16 and anti‐HPV‐18 GMTs were 1271.1 EU/mL (1135.8–1422.6) and 710.0 EU/mL (628.6–801.9), respectively, and remained above anti‐HPV‐16/18 GMT plateaus associated with sustained protection in a previous efficacy study 24 (Fig. 3).

### Safety

The safety outcomes are presented in Table 3. A total of 126 (2.1%) women reported at least one SAE in the current follow‐up (Table 3). One SAE (epigastric pain) was considered by the investigator as possibly related to vaccination (the group assignment is not reported to avoid unblinding). The most common SAE was appendicitis, reported by 16 (0.3%) women. Five women experienced at least one SAE leading to premature study discontinuation, but none of these SAEs were considered by the investigator as possibly related to vaccination. One fatal SAEs (suicide) assessed by the investigator as not related to vaccination was reported during the study (the group assignment is not reported to avoid unblinding).

**Table 3:** Safety outcomes at the time of event‐triggered analysis with a mean follow‐up of approximately 57 months (TVC)

	Vaccine*N *=* *3026	Control*N *=* *3025	Total*N *=* *6051
	*n* (%)		
Serious adverse events	50 (1.7)	76 (2.5)	126 (2.1)
Medically significant conditions	181 (6.0)	180 (6.0)	361 (6.0)
New‐onset of chronic diseases	8 (0.3)	12 (0.4)	20 (0.3)
New‐onset of autoimmune diseases	2 (0.1)	2 (0.1)	4 (0.06)

TVC, total vaccinated cohort; Vaccine, women who received up to three doses of the HPV‐16/18 AS04‐adjuvanted vaccine; Control, women who received up to three doses of aluminium hydroxide; *N*, number of evaluable women in each group; *n* (%), number/percentage of participants with the event.

A total of 361 women (6.0%) reported at least one medically significant AE (Table 3); the most common was pelvic inflammatory disease (22 women; 0.4%).

Overall, NOCDs and NOADs were rarely reported (0.3% and 0.06% of women, respectively) (Table 3). The most common NOCD was allergic dermatitis, reported by three women (0.1%) from the vaccine group and four women (0.1%) from the control group. Among the four NOADs reported, psoriasis was reported by two women (one in each group), and hyperthyroidism and VIIth nerve paralysis were reported in one woman each (blinded data). None of the NOADs reported were assessed by the investigator to be causally related to vaccination.

A total of 1595 pregnancies were reported (the total numbers per group are not reported to avoid unblinding; Table S2). The majority of pregnancies (74.0%) resulted in live infants with no apparent congenital anomalies, while 17.6% of pregnancies with no apparent congenital anomalies were electively terminated. Two women (0.1%) reported stillbirths with no apparent congenital anomalies. Nine pregnancies (0.6%) resulted in an offspring with congenital anomalies assessed by the investigator as unlikely related to the study vaccine administration; five (0.3%) resulted in live infants, three (0.2%) were electively terminated, and one (0.1%) resulted in a stillbirth. As no pattern in the nature of the congenital anomalies was noticed, the pregnancy data will remain blinded to treatment allocation until the study end. Fifty‐one (3.2%) pregnancies were ongoing at the time of the current analysis.

A total of 25 pregnancies were reported around the time of vaccination. Eight pregnancies (0.5%) resulted in birth of a live infant with no congenital anomaly and 14 (0.9%) without any apparent congenital anomaly were electively terminated (Table S2). There were no pregnancies with congenital anomaly around the time of vaccination.

## Discussion

### Main findings

In China, cervical cancer is the second most frequent cancer, following breast cancer, among women aged 30–44 years, and the third most common, following thyroid cancer and breast cancer, in women below the age of 30 34. With 137 billion people, China constitutes one‐fifth of the global population. Thus, the cervical cancer burden in China has a substantial effect on global estimates of the cervical cancer burden; the estimated 98,900 new cases reported in China in 2015 account for 18.7% of the global incidence 11. Although in 2009, China has initiated a government‐supported national cervical cancer registry and screening programs targeting rural population of women aged 35–64 years, cervical cancer remains a major health care concern, due to low screening coverage, limited financial resources, and lack of preventive HPV vaccination programs.

This study of the HPV‐16/18 AS04‐adjuvanted vaccine is to the best of our knowledge the first large controlled clinical study of vaccination efficacy against HPV conducted in China. We previously reported a high VE (94.2% [62.7–99.9]) against HPV‐16/18‐associated CIN1+ and/or 6M PI within 24 months following last vaccine dose 30. However, only three cases of CIN2+ were observed in the previous analysis due to the short follow‐up time (approximately 21 months post‐dose 1 in the ATP‐E). CIN2+ has been the traditional surrogate to cervical cancer for evaluation of VE, and has been defined as a primary endpoint in two large previous efficacy trials: the global randomized, double‐blind, controlled PApilloma TRIal against Cancer In young Adults (PATRICIA) 17, 20, 21 and the community‐based trial in Costa Rica 29, 35. However, to obtain enough CIN2+ cases for assessment, a large sample size and extensive follow‐up are required 36. Therefore, the follow‐up time of this study was extended up to 72 months post‐dose 1 to allow further evaluation of VE for this endpoint.

A high VE (87.3%; 5.3–99.7) against CIN2+ associated with HPV‐16/18 was observed in both ATP‐E and TVC‐E cohorts in women with seronegative status at baseline. One CIN2+ case was observed in the vaccine group; however, the assessment of real causal association was difficult due to the multiple oncogenic HPV infections that were detected in both the lesion and the preceding cytological specimens of this case. A similar phenomenon was also observed in the PATRICIA trial; in 28.6% of the 256 CIN2+ cases associated with HPV‐16 and/or HPV‐18, DNA from nonvaccine HR‐HPV types was also detected in the final event‐driven analysis 18.

The overall efficacy results of this study are in line with the previous efficacy trials conducted in similar age populations 17, 18, 19, 20, 21, 22, 25, 26, 27, 28. The mean follow‐up in this study (approximately 52 months post‐dose 3 in the ATP‐E and approximately 57 months post‐dose 1 in the TVC‐E) was longer than the mean follow‐up in the final end‐of‐study analysis of the PATRICIA trial (43.7 months in the TVC) 17. As the efficacy estimates appeared to increase with longer duration of the follow‐up in the PATRICIA trial 17, 20, 21, the efficacy results of the current follow‐up indicate that the HPV‐16/18 AS04‐adjuvanted VE against PI and cervical lesions is sustained in young Chinese women. In addition, the long‐term follow‐up of this study allowed for evaluation of the cross‐protective VE against nonvaccine HPV types. A significant VE against 6M PI and 12M PI was shown for combined HPV‐31/33/45 (TVC‐E; 41.9% [15.8–60.4] and 43.1% [0.2–68.3], respectively); evidence of cross‐protection against PI with a different combination of nonvaccine HPV types (HPV‐31/33/45/51 or HPV‐31/33/45/52/58) was also shown in the PATRICIA trial 18, 21. However, in our study, the sample size was smaller than in the PATRICIA trial, which might explain why statistically significant VE was not reached for all the nonvaccine types, for which cross‐protection was observed in the global PATRICIA study. In addition, for some nonvaccine HPV types, for example, HPV‐45 or HPV‐58, VE against incident infection was higher than VE against 6M PI. This could possibly be explained by masking of some nonvaccine types in the control group in case of multiple infections due to the PCR method used, resulting in a bias against the vaccine 37.

The anti‐HPV‐16/18 antibody kinetics were also in line with previous observations 24, 38, 39, 40. anti‐HPV‐16/18 levels at M24 remained several folds above the GMT plateaus associated with sustained protection at M45–50 in a previous efficacy study 24. High and sustained antibody titers obtained through vaccination are considered predictive of long‐term protection against oncogenic HPV infections and CIN through a mechanism involving transudation of vaccine‐induced neutralizing antibodies across the cervical epithelium to the site of HPV infection 41, 42, 43, 44. The high and sustained anti‐HPV‐16/18 antibody responses observed in this study indicate that the HPV‐16/18 AS04‐adjuvanted vaccine can be expected to provide strong and long‐term protection against HPV‐16/18 infections in Chinese women.

The proportions of women with SAEs, MSCs, NOCDs, and NOADs were similar in the vaccine and control group, consistent with finding of the previous studies of the HPV‐16/18 AS04‐adjuvanted vaccine in similar age group 19, 21, 22, 45. Only one SAE related to vaccination was reported. The incidence of MSCs among vaccinated women (6.0%) was similar to that previously reported in vaccinated women aged 15–25 years from the HPV‐023 study (8.1%) 22, and the incidence of NOCD and NOADs was very low (0.3% and 0.1%, respectively), also in line with previous reports (0.0–3.0% and 0.0–1.0%, respectively) 21, 22, 45. The incidence of congenital anomalies in offspring of all pregnant women throughout the study was comparable with that observed in the large global clinical trial 17, and was low compared with the reported incidence of congenital anomalies worldwide and in China 46, 47, 48.

The study design was similar to other large trials evaluating the efficacy of the HPV‐16/18 AS04‐adjuvanted vaccine in terms of population, vaccination schedule, clinical management for abnormal cytology, colposcopy referral, and HPV DNA testing 20, 21, 35, 49. However, in our study, the primary endpoint was prospectively defined as prevention of histopathologically confirmed CIN1+ and/or 6M PI associated with HPV‐16/18, with CIN2+ as a secondary endpoint, whereas the PATRICIA trial and the Costa Rica vaccine trial defined CIN2+ as the primary endpoint. Nevertheless, VE in a global population was first demonstrated in the PATRICIA trial, and the results of this study in Chinese women are consistent with those from the global study, allowing the “bridging” of the global efficacy results to the Chinese population.

### Strengths and limitations

This study is the first long‐term large‐scale clinical trial of HPV vaccination conducted in China. Additional strengths of the study include the facts that the clinical examination and diagnosis were performed according to a unified standard and the HPV testing was performed in a high‐quality control central laboratory. In this study, local Chinese laboratories were used which were different from the central laboratories in the other efficacy trials. The consistency of the efficacy results between the Chinese and global populations while using different laboratories for diagnosis of the efficacy endpoints demonstrate the robustness of these efficacy findings.

A potential limitation of the study is that all women included in this study were sexually active (majority were married) at the time of study entry due to ethical and cultural considerations, and there were more women who were closer to the upper age limit (mean age 23 years at vaccination). Another limitation is inclusion of women from only one of the seven geographic regions in China, which might not fully reflect the overall Chinese population.

## Conclusions

The results of this follow‐up study in young Chinese women show that the HPV‐16/18 AS04‐adjuvanted vaccine is efficacious against cervical infections, abnormal cytology, and lesions associated with HPV‐16/18. In addition, cross‐protective VE against PI with HPV‐31/33/45 was consistent with that previously reported in the global PATRICIA efficacy trial. The vaccine induced high and sustained anti‐HPV‐16 and anti‐HPV‐18 antibody responses that were comparable with those observed in global trials, and had a clinically acceptable safety profile.

## Conflict of Interest

All authors have completed the Unified Competing Interest form at http://www.icmje.org/coi_disclosure.pdf and declare the following potential conflicts of interest: The institutions of Fang‐Hui Zhao, Shang‐Ying Hu, Ying Hong, Yue‐Mei Hu, Xun Zhang, Yi‐Ju Zhang, Qin‐Jing Pan, Wen‐Hua Zhang, Cheng‐Fu Zhang, Xiaoping Yang, Jia‐Xi Yu, Jiahong Zhu, Yejiang Zhu, Feng Chen, Qian Zhang, Hong Wang, Changrong Wang, Jun Bi, Shiyin Xue, Lingling Shen, Yan‐Shu Zhang, Feng‐cai Zhu received grants/investigator fees from the GSK group of companies for the conduct of this study. Fang‐Hui Zhao, Feng‐cai Zhu, and Yue‐Mei Hu received support for travel to meetings related to the study from the GSK group of companies. Yunkun He, Haiwen Tang, Naveen Karkada, Pemmaraju Suryakiran, Dan Bi, and Frank Struyf are employees of the GSK group of companies. Haiwen Tang, Dan Bi, and Frank Struyf own stock options and shares in the GSK group of companies.

## Supporting information


**Table S1.** Vaccine efficacy against cervical intraepithelial neoplasia and persistent infection associated with HPV‐16 and/or HPV‐18 in women who were HPV DNA‐negative at baseline for the corresponding HPV type, regardless of initial serostatus (ATP‐E and TVC‐E).
**Table S2.** Pregnancy outcomes (TVC).
