# Supplementary material for: Efficacy, immunogenicity, and safety of the HPV‐16/18 AS04‐adjuvanted vaccine in Chinese women aged 18–25 years: event‐triggered analysis of a randomized controlled trial
Source: Cancer Med. 2016 Dec 20;6(1):12–25. doi: 10.1002/cam4.869 (PMC5269697; doi:10.1002/cam4.869)
Supplement: Supplementary file 1 — Table S1. Vaccine efficacy against cervical intraepithelial neoplasia and persistent infection associated with HPV‐16 and/or HPV‐18 in women who were HPV DNA‐negative at baseline for the corresponding HPV type, regardless of initial serostatus (ATP‐E and TVC‐E). Table S2. Pregnancy outcomes (TVC). [file CAM4-6-12-s001.docx]

**Supplementary Table S1.** Vaccine efficacy against cervical intraepithelial neoplasia and persistent infection associated with HPV-16 and/or HPV-18 in women who were HPV DNA-negative at baseline for the corresponding HPV type, regardless of initial serostatus (ATP-E and TVC-E)

| **HPV-16/18 Endpoint** | **ATP-E** | | |  | **TVC-E** | | |
| --- | --- | --- | --- | --- | --- | --- | --- |
|  | **Vaccine** | **Control** | **% VE (95% CI)** |  | **Vaccine** | **Control** | **% VE (95% CI)** |
|  | **N/n** | **N/n** |  |  | **N/n** | **N/n** |  |
| CIN2+ | 2805/1 | 2802/10 | 90.0 (29.4–99.8) |  | 2853/2 | 2857/12 | 83.2 (24.7–98.2) |
| CIN1+ | 2805/1 | 2802/18 | 94.4 (64.7–99.9) |  | 2853/3 | 2857/21 | 85.7 (51.9–97.3) |
| CIN1+/6M PI | 2805/2 | 2802/78 | 97.5 (90.5–99.7) |  | 2853/7 | 2857/99 | 93.0 (85.1–97.3) |
| 6M PI | 2759/2 | 2744/70 | 97.2 (89.4–99.7) |  | 2835/5 | 2839/90 | 94.5 (86.7–98.3) |
| 12M PI | 2699/1 | 2706/37 | 97.3 (84.0–99.9) |  | 2799/4 | 2795/49 | 91.9 (77.8–97.9) |
| ATP-E, according-to-protocol cohort for efficacy; TVC-E, total vaccinated cohort for efficacy; Vaccine, women who received up to 3 doses of the HPV-16/18 AS04-adjuvanted vaccine; Control, women who received up to 3 doses of aluminium hydroxide; N, number of participants included in each group; n, number of cases; VE, vaccine efficacy; 95% CI, 95% confidence interval; CIN2+, cervical intraepithelial neoplasia grade 2 and above; CIN1+/ 6M PI, cervical intraepithelial neoplasia grade 1 and above and/or 6-month persistent infection  CIN2+ was defined as CIN2, CIN3, low-grade cervical glandular intraepithelial neoplasia (LCGIN), high-grade cervical glandular intraepithelial neoplasia (HCGIN), adenocarcinoma in-situ (AIS) or invasive cervical cancer.  CIN1+ was defined as CIN1, CIN2, CIN3, LCGIN, HCGIN, AIS or invasive cervical cancer.  6M PI with HPV-16/18 was defined as at least 2 positive HPV DNA PCR assays for the same viral genotype with no negative DNA sample between the 2 positive DNA samples, over at least 150 days. | | | | | | | |

**Supplementary Table S2.** Pregnancy outcomes (TVC)

| **Pregnancy outcome** |  |
| --- | --- |
| **Total number of pregnancies** | **1595** |
| Live infant: no apparent congenital anomaly, n (%) | 1181 (74.0) |
| Pregnancies with congenital anomaly, n (%) | 9 (0.6) |
| Live infant | 5 (0.3) |
| Elective termination | 3 (0.2) |
| Stillbirth | 1 (0.1) |
| Elective termination: no apparent congenital anomaly, n (%) | 281 (17.6) |
| Ectopic pregnancy, n (%) | 14 (0.9) |
| Spontaneous abortion: no apparent congenital anomaly, n (%) | 29 (1.8) |
| Stillbirth: no apparent congenital anomaly, n (%) | 2 (0.1) |
| Lost to follow-up, n (%) | 28 (1.8) |
| Pregnancy ongoing, n (%) | 51 (3.2) |
| **Number of pregnancies around vaccination*, n (%)** | **25 (1.6)** |
| Live infant: no apparent congenital anomaly, n (%) | 8 (0.5) |
| Elective termination: no apparent congenital anomaly, n (%) | 14 (0.9) |
| Ectopic pregnancy, n (%) | 1 (0.1) |
| Spontaneous abortion: no apparent congenital anomaly, n (%) | 2 (0.1) |
| n (%), number/percentage of pregnancies with the event  The total numbers per group are not shown to avoid unblinding.  *Pregnancies around vaccination were defined as pregnancies of women for whom the last menstrual period occurred between 30 days before and 45 days after vaccination. | |
